# Resection of large sacral tumors: a report of 4 cases

**Published:** 2012-11

**Authors:** Majid Reza Farrokhi, Hormoz Nouraei, Seyed Vahid Hosseini

**Affiliations:** ^*a*^Shiraz Neurosciences Research Center, Neurosurgery Department, Shiraz University of Medical Sciences, Shiraz, Iran.; ^*b*^Department of Orthopaedic surgery, Shiraz University of Medical Sciences, Shiraz, Iran.; ^*c*^Department of Surgery, Shiraz University of Medical Sciences, Shiraz, Iran.

## Abstract

Tumors of the sacrum and related neurological and pelvic structures are rare pathologic conditions.

Sacral tumors are a diagnostic challenge because patients often present with nonspecific complaints such as low back pain, sacrococcygeal joint pain, referred lag and buttock pain. Because of diagnostic and radiographic challenges, the detection of sacral mass can be delayed which complicates any prospect surgical intervention as tumors can be quite large at the time of treatment. Furthermore, the complex local anatomy of the sacrum and presence of numerous other structures make the resection procedure even more sophisticated and necessitate adoption of a multidisciplinary approach towards the treatment. The present study discuses the diagnosis, treatment and complication of 4 cases with large sacral mass.

**Keywords:**

Resection, Sacral, Tumor


					Volume 4, Suppl. 1 Nov 2012
Publisher: Kermanshah University of Medical Sciences
URL: http://www.jivresearch.org
Copyright:
Congress of Iranian Neurosurgeons, 14-16 November 2012, Kermanshah, Iran
Paper No. 65
Resection of large sacral tumors: a report of 4 cases
Majid Reza Farrokhi a, *
, Hormoz Nouraei b
, Seyed Vahid Hosseini c
a
Shiraz Neurosciences Research Center, Neurosurgery Department, Shiraz University of Medical Sciences, Shiraz, Iran.
b
Department of Orthopaedic surgery, Shiraz University of Medical Sciences, Shiraz, Iran.
c
Department of Surgery, Shiraz University of Medical Sciences, Shiraz, Iran.
Abstract:
Tumors of the sacrum and related neurological and pelvic structures are rare pathologic conditions.
Sacral tumors are a diagnostic challenge because patients often present with nonspecific complaints such as low back
pain, sacrococcygeal joint pain, referred lag and buttock pain. Because of diagnostic and radiographic challenges,
the detection of sacral mass can be delayed which complicates any prospect surgical intervention as tumors can be
quite large at the time of treatment. Furthermore, the complex local anatomy of the sacrum and presence of
numerous other structures make the resection procedure even more sophisticated and necessitate adoption of a
multidisciplinary approach towards the treatment. The present study discuses the diagnosis, treatment and
complication of 4 cases with large sacral mass.
Keywords:
Resection, Sacral, Tumor
* Corresponding Author at:
Majid Reza Farrokhi: Professor of Neurosurgery, Shiraz Neurosciences Research Center, Neurosurgery Department, Shiraz University of Medical
Sciences, Shiraz, Iran, Mob: 09171114472Tel: +98711-6234508, E-mail: farokhim@sums.ac.ir , farrokhi_drmr@yahoo.com, (Farrokhi MR.).

				

